# Contrasting fine-scale genetic structure of two sympatric clonal plants in an alpine swampy meadow featured by tussocks

**DOI:** 10.1371/journal.pone.0209572

**Published:** 2018-12-21

**Authors:** Yu Ning, Gao-Jie Wu, Hua Ma, Ju-Lan Guo, Man-Yin Zhang, Wei Li, Yi-Fei Wang, Suo-Lang Duoerji

**Affiliations:** 1 Institute of Wetland Research, Chinese Academy of Forestry, Beijing, China; 2 Zoige Alpine Wetland Ecosystem Research Station, Zoige, Sichuan, China; 3 Key Laboratory of Wetland Services and Restoration, Chinese Academy of Forestry, Beijing, China; 4 Administration of Zoige Wetland National Nature Preserve, Zoige, Sichuan, China; University of North Carolina at Greensboro, UNITED STATES

## Abstract

Tussocks are unique vegetation structures in wetlands. Many tussock species mainly reproduce by clonal growth, resulting in genetically identical offspring distributed in various spatial patterns. These fine-scale patterns could influence mating patterns and thus the long-term evolution of wetland plants. Here, we contribute the first genetic and clonal structures of two key species in alpine wetlands on the Qinghai–Tibet Plateau, *Kobresia tibetica* and *Blysmus sinocompressus*, using > 5000 SNPs identified by 2b-RAD sequencing. The tussock-building species, *K*. *tibetica*, has a phalanx (clumping) growth form, but different genets could co-occur within the tussocks, indicating that it is not proper to treat a tussock as one genetic individual. Phalanx growth does not necessarily lead to increased inbreeding in *K*. *tibetica*. *B*. *sinocompressus* has a guerilla (spreading) growth form, with the largest detected clone size being 18.32 m, but genets at the local scale tend to be inbred offspring. Our results highlight that the combination of clone expansion and seedling recruitment facilitates the contemporary advantage of *B*. *sinocompressus*, but its evolutionary potential is limited by the input genetic load of the original genets. The tussocks of *K*. *tibetica* are more diverse and a valuable genetic legacy of former well-developed wet meadows, and they are worthy of conservation attention.

## 1. Introduction

Vegetation is of fundamental importance to alpine ecosystems through processes such as water retention and evapotranspiration [1, 2]. The genetic diversity of plant species constitutes an essential component of biodiversity, as it serves as the basis for evolution, especially in the face of emerging challenges such as climate change and over-grazing [3]. However, the genetic structure of alpine plants is complicated by the prevalence of clonal growth. Clonal growth is an asexual reproductive strategy that is favored in harsh habitats, such as tundra, desert and alpine areas [4, 5]. During clonal growth, genetically identical offspring (ramets) are produced vegetatively. Ramets usually remain connected by spacers such as rhizomes or solons to form an entire clone (genet). This reproductive mode provides the plants with the advantages of physiological integration and labor division to persist in extreme environments [6, 7]. Clonal growth also has a profound effect on genetic diversity and evolutionary potential because it affects the mating patterns of a plant population. The impacts rely on two main aspects: clone size and spatial arrangement. There have been debates about the effect of the size and distribution of clones on the genetic structure of the clonal plant population (reviewed by Vallejo-Marin *et al*. [8]). Generally, the clonal population is comprised of clones with uneven sizes and frequencies, so a few large clones tend to contribute the main portion of the genetic component, making the effective population size smaller than the apparent census population [9]. Additionally, the spatial arrangement of genets differs according to the pattern of clonal growth. Short spacers result in a clumped distribution of ramets (phalanx form), while longer spacers can place ramets in various directions over long distances (guerrilla form). The former often produce a separated distribution of genets, whereas the latter could present an intermingled pattern. It has been reported that the phalanx growth form tends to increase the chances of geitonogamous selfing, particularly with increasing genet size, and consequently increases the risk of inbreeding depression [10–12]. Nevertheless, clonal reproduction is also associated with mass flowering, which increases the opportunity for pollinator visiting. Flowers on the periphery of large clones may receive outcross pollen more easily than smaller clones [13]. Generally, the greatest genetic impact of clonality often occurs at fine spatial scales within populations, due to the limited dispersal capability of asexual reproduction. It is crucial to determine the spatial patterns of clones and the impact of clonal growth on fine-scale genetic structure if any effective conservation management is to be issued.

The presence of tussocks is a feature of the topography in wetland systems. In water logged sites, roots of the builder species capture and retain sediment on which plants continue to grow and develop tussocks [14]. Mature tussocks may have expanded basal areas and bear many cohabitant species. Facilitation may be the underlying mechanism that promotes the coexisting of tussock species. By providing some facilitative effects (e.g., grazing prevention, warmth trap and physical stress relief), tussocks act as fine-scale shelters for sympatric species [15, 16]. Previous studies have demonstrated that individuals in tussocks tend to have more sexual reproduction events [15, 17]. This phenomenon is of vital importance because generatively reproducing individuals inside tussocks could serve as seed sources and make a critical contribution to the genetic pool of ecosystems preferring clonal growth. However, it is currently unknown whether coexisting species in tussocks have the same exact genetic structure or mating pattern. Furthermore, individuals from the same tussock are often presumably treated as belonging to the same clone (but see *Carex sempervirens* [18]), which may not be true. Arbitrary clone assignment could result in a biased estimation of the mating pattern and gene flow process. Knowledge of clonality is essential if any inference is to be made about the genetic structure of tussock wetland species.

In wetlands of the Qinghai–Tibet Plateau (QTP), such as swampy meadows, waterlogged areas, and river margins, the vegetation is typically characterized by *Kobresia tibetica* Maxim, which is a tussock-building perennial with clumped and erect stems [19]. *Kobresia* is the key species in the alpine ecosystem of the QTP. *Kobresia* pastures in the eastern Tibetan highlands occupy 450000 km^2^ and form the world’s second largest alpine ecosystem [20]. However, the recent increase in surface soil temperatures and anthropogenic disturbance has led to a deterioration of *K tibetica* swamps and retrogressive succession. A commonly seen successional hygrophyte is *Blysmus sinocompressus* Tang&F.T.Wang. *B*. *sinocompressus* appears at the early stage of retrogressive succession and gradually replaces *K*. *tibetica* as the degradation proceeds [20–22]. *B*. *sinocompressus* is low in height compared with *K*. *tibetica*. This species often establishes a continuous population, indicating a possible spreading growth form. These two species both have mixed reproductive strategies. They mainly rely on clonal growth. Limited sexual reproduction occurs when environmental conditions are optimal [23, 24]. Previous studies have evaluated the genetic diversity of both species at the regional scale [25, 26]. The results have shown that, for both species, limited sexual reproduction appears capable of maintaining the genetic diversity level, and more genetic variation resides within populations rather than among populations. These results indicate that fine-scale genetic structure may exist and play an important role in the gene flow process, which has not yet been explored. Moreover, the specific clonal structure of both species is currently unknown, so the effect of clonality on population genetics and tussock succession remains poorly understood.

Here, we present the first comparative study on the fine-scale genetic structure of these two clonal plants, *K*. *tibetica* and *B*. *sinocompressus*, which are typical species in the alpine wetland of the eastern part of the Qinghai–Tibet Plateau. The aim of the study was to determine the following: (1) the specific clonal structures of these two species; (2) the spatial range on which clonality affects the genetic pattern; and (3) the fine-scale genetic structure and diversity that could help explain the successional process of tussock swamps. Specifically, we designed a specialized sampling scheme, estimated the genotypic diversity and inbreeding level and determined the spatial architecture of the clonal lineage using single nucleotide polymorphism (SNP) loci generated by 2b-RAD sequencing. Spatial autocorrelation analyses were implemented at both the ramet and genet levels to assess the impact of clonality on the fine-scale genetic structure for each species. We anticipate that the findings could be helpful in the conservation of alpine wetland plants and the sustainable management of swampy meadows featured by tussocks.

## 2. Materials and methods

### 2.1. Study area and species

This study was conducted at the Zoige wetland in the eastern margin of the Qinghai–Tibet Plateau. The altitude in this area ranges from 3400–3600 m. The mean annual temperature is approximately 0.6–1.0°C. The majority of the precipitation occurs in summer, 580–860 mm annually. Perennial herbaceous species dominate the regional vegetation, of which Cyperaceous species account for more than 80% [27]. Our sampling stand was set in a natural wet meadow (33°47′53.59″N, 102°57′33.74″E) that is approximately 25 km north of Zoige County. This meadow is mainly used as a herd pasture with no specific management regime except fencing at the boundary. The landscape of the stand is generally flat in terrain with scattered distributed tussocks. The total vegetation coverage is approximately 95% by observation.

**Fig 1:**
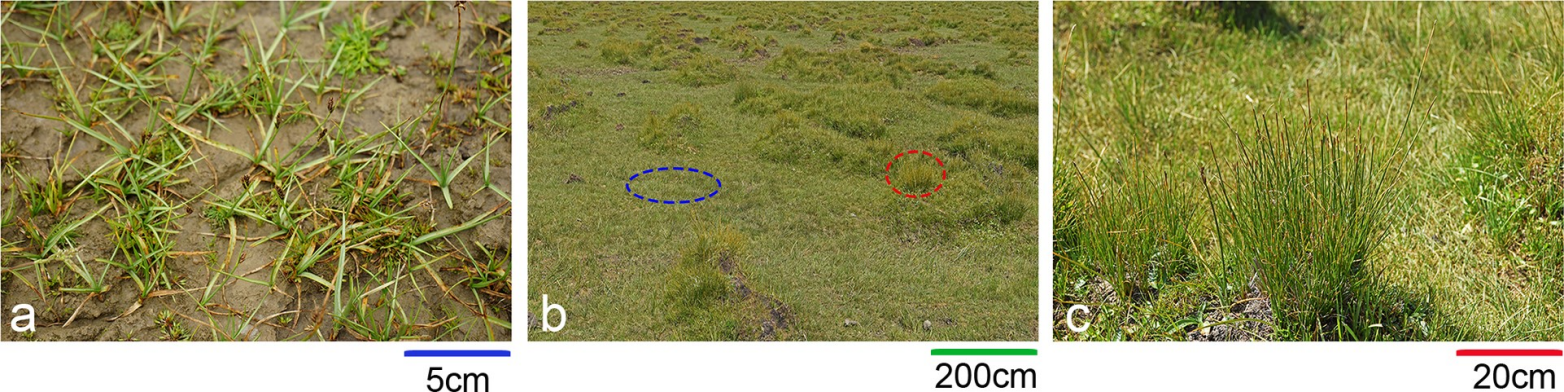
Community landscape and species. a. *Blysmus sinocompressus*; b. community view with the blue circle indicating *B*. *sinocompressus* and the red circle indicating *K*. *tibetica*; c. *Kobresia tibetica*. The different bars beneath each picture scale with the actual range.

The vegetation is mainly composed of two species: *Kobresia tibetica* Maxim. and *Blysmus sinocompressus* Tang & F. T. Wang. Both species are typical endemic hygrophytes in the Qinghai–Tibet Plateau, often co-occurring at riverbeds, stream margins, swampy meadows, etc. Their morphological traits differ greatly. *K*. *tibetica* has dense, rigid and erect culms [28]. It is usually the builder species of local tussocks. *B*. *sinocompressus* has dwarf culms with brown to purplish leaf sheaths at the base [24]. This plant often appears even and continuous on the landscape with no apparent aggregation. The growing period of both species usually ranges from May until dormancy commences in October. The flowering and fruiting phenology of these two species lasts from May to September. However, the seed germination rate of both species has been found to be low in natural conditions, ranging from 0 to 13% [25, 29]. Vegetative reproduction has been reported to be ubiquitous, indicating the significant importance of clonal growth in their life histories [30]. Fig 1 shows a brief description of the community and species.

### 2.2. Stand design and sample collection

**Fig 2:**
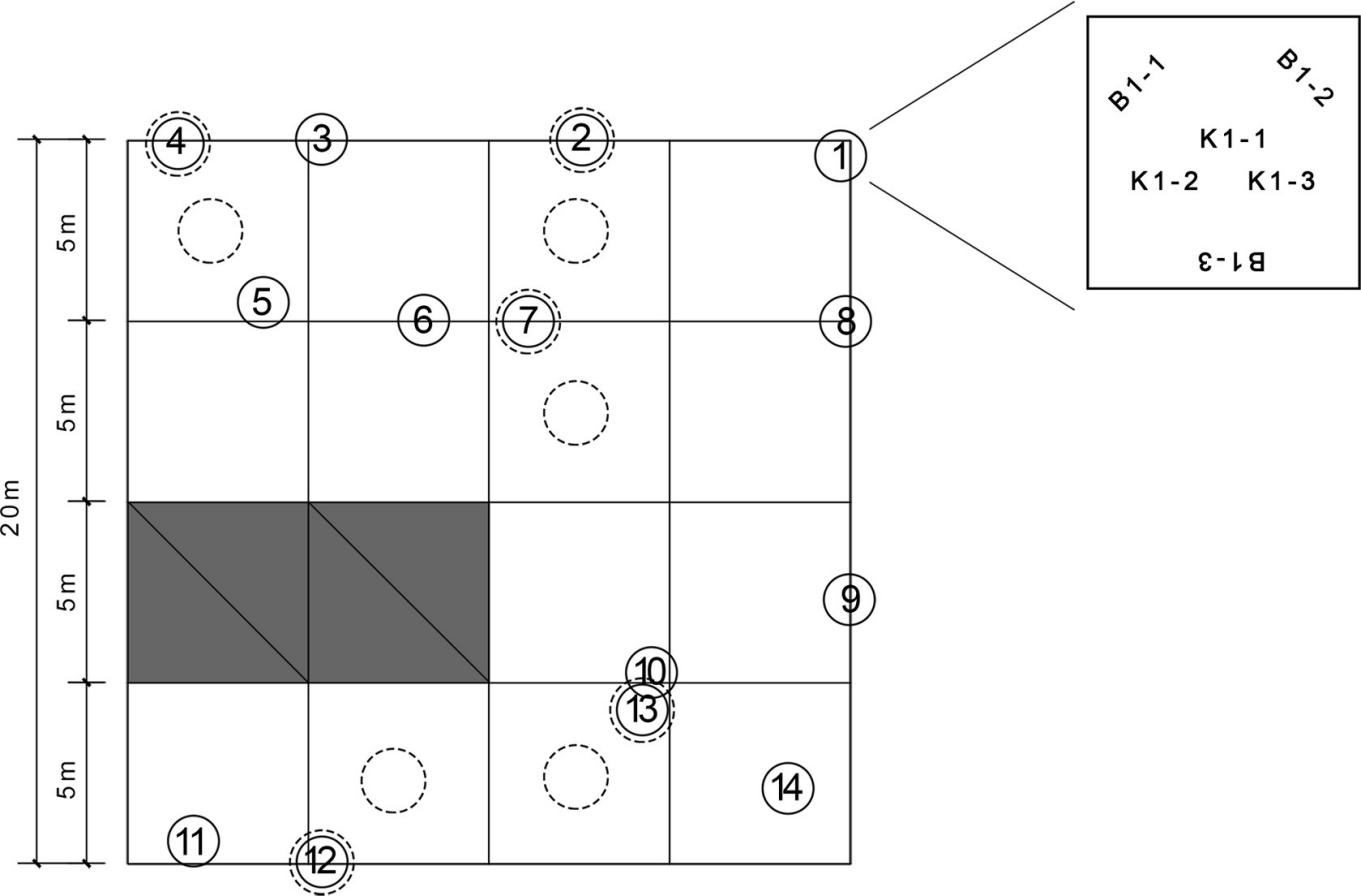
Stand design and sampling scheme. The solid circles with numbers represent the sampled tussocks. The dashed circles indicate where the soil profiles were taken. The inset shows where the three samples of *K*. *tibetica* and the three samples of *B*. *sinocompressus* were taken at each tussock.

All the samples were collected from one 20 m×20 m stand. The Administrative Bureau of Zoige Wetland National Nature Preserve approved this fieldwork. No endangered/ protected species were involved in this study. We chose a square plot style to collect the samples to minimize possible edge effects, as recommended by Arnaud-Haond *et al*. [31]. Some adjustments were made to facilitate the sampling of the tussocks and to compare the two species. Specifically, the sampling stand was divided into 16 subplots with equal sizes of 5 m×5 m. In each subplot, we chose one tussock and scaled it to the boundary of the subplot to generate the spatial coordinates (see S1 Table). At each tussock, three randomly chosen culms of *K*. *tibetica* and *B*. *sinocompressu*s were clipped to the base. Two subplots were not sampled due to the absence of *K*.*tibetica* tussocks; thus, 84 plant samples were collected in total, 42 samples for each species. The community demography was investigated by setting a 50 cm×50 cm quadrat at each sampling tussock. The abundance, height and coverage of these two species were measured. To compare the fine-scale habitat conditions for the two species, soil profiles were sampled in a pairwise manner. The soil profiles of *K*. *tibetica* were taken within the tussocks, while the soil profiles of *B*. *sinocompressu*s were taken in the gap between the tussocks. Five soil profiles were made for each species. Each soil profile consisted of six layers from the surface to a depth of 1 m at a 20 cm interval. All the soil samples were analyzed for organic matter, available nitrogen, available potassium, available phosphorus, pH, electrical conductivity, saturated hydraulic conductivity and water content. The assaying procedures followed those of Carter and Gregorich [32]. Fig 2 demonstrates the sampling scheme.

### 2.3. DNA extraction and genotyping

All the plant samples were collected with caution to prevent extraneous DNA interference. After inspection of their validity, the samples were preserved with silica and delivered to the lab. DNA was extracted using the Plant Genomic DNA Kit (Tiangen Biotech Co., Beijing, China). The sequencing was completed using an Illumina HiSeq X Ten platform. The 2b-RAD libraries [33] were constructed following the method of the serial sequencing of the isolength RAD tags. This method allows the preparation of five concatenated isoRAD tags for Illumina paired-end sequencing [34]. Adaptors (5’-NNN-3’) were used to cohere the digested products (detailed information of the adaptors and primers is listed in S2 Table). The raw reads were trimmed to remove the adaptor sequences, and the 3-bp terminal positions of each read were eliminated. Reads with no restriction sites or ambiguous bases (N), low-quality positions (>20 nucleotide positions with a Phred quality score < 20), or long homopolymer regions (>8%) were discarded. The high-quality reads of each sample were aligned using the SOAP2(version 2.21) program following the protocols by Li *et al*. [35]. A maximum of two mismatches (–v 2) was allowed for each read, and those that mapped onto more than one position in the genomic reference sequence were excluded (–r 0). The match mode was set to “find the best hits” (–M 4). Because prior genome information is limited for both species, we used a modified reference approach for the genotyping. Taking *K*. *tibetica* for instance, we randomly chose five samples. The enzyme reads (reads with restriction sites for *Bsa*XI) of these samples were clustered to construct a reference sequence using uSTACKS (version 1.34). Then, the enzyme reads of all the remaining samples of this species were referenced to this sequence using SOAP2 (version 2.21), and the SNPs were obtained based on a maximum likelihood algorithm and filtered by the RADtyping program [36]. The same procedure was also applied to *B*. *sinocompressus*. Finally, 41 samples of *K*. *tibetica* and 39 samples of *B*. *sinocompressu*s were successfully sequenced and genotyped.

### 2.4. Clone assignment and spatial structure

Most of the genetic analysis described below was carried out using the computer and statistical language R with various packages [37]. We acquired unique multilocus genotypes (MLGs) from the 2b-RAD genotyping. To characterize the genetic diversity and clonal structure correctly, the distinct genets had to first be identified. The package *poppr* (version 2.6) was implemented to assign clonal membership [38, 39]. The main procedure consisted of creating a genetic distance matrix, finding the threshold and collapsing the different MLGs into genets. The minimum genetic distance to distinguish the different MLGs (i.e., threshold) was calculated using the *cutoff_predictor* function. Then the threshold was conveyed to the *Mlg*.*fliter()* function to assign a clone affiliation to each ramet. Based on the results of the clone identification, the clone size, richness and distribution status were evaluated at the scale of the whole stand. Clonal richness was calculated as (G-1)/ (N-1). We analyzed the genotype diversity using the Shannon-Wiener index (H) and Stoddard and Taylor's index (G). Both indexes measure genotypic diversity by combining richness and evenness. If all the genotypes are equal in abundance, the value of G will be the number of MLGs and the value of H will be the natural log of the number of MLGs [40]. G and H were used in combination because they are complementary in their weighting of abundant or rare MLGs. Evenness (E) was calculated utilizing both H and G, resulting in a ratio of the number of abundant genotypes to rare genotypes [41]. G, H and E were calculated using the *diversity ()* function in package *vegan* (version 2.0) [42]. All the identified MLGs were mapped to assess the spatial arrangement of the clonal patches. We performed a spatial autocorrelation analysis at both the ramet level and the genet level following the suggestion of Binks *et al*. [9]. The ramet level analysis included all the sampled individuals. The genet level analysis kept only one ramet per MLG. The calculation procedure was carried out using the *spline*.*correlog()* function in *ncf* package(version 1.2)[43], with the genetic distance matrix created using the *dis*.*bitwise()* function in *poppr* and the spatial coordinates generated from field records. Moran’s I was calculated, and 1000 resamples were implemented to find the bootstrap distribution.

### 2.5. Genetic diversity and evolutionary relationship

To avoid the influence of clonality on the genetic diversity estimation, we removed the replicates from each genet and continued the analyses using a single copy of each unique genotype. We used Genepop (version 4.7) [44] to calculate the allelic richness, expected heterozygosity, observed heterozygosity, and the inbreeding coefficients of both species at the stand scale. To evaluate the extent of the differentiation, an analysis of molecular variance (AMOVA) was implemented using the *amova ()* function in the *pegas* [45] package to evaluate the extent of genetic variation within and between the sampled tussocks. The relationship between the different MLGs was inspected using minimum spanning networks (MSN) with reticulation. Reticulated MSN reduces the complexity of a distance matrix and allows the population structure to be more readily detectable. It is a more suitable tool than bifurcating trees for clonal organisms where many of the connections between the samples are equivalent [38]. The MSN result was visualized using the *imsn()* function in the *poppr* package.

## 3. Results

### 3.1. Summary of the community topology and environmental factors

The results of the demography investigation showed that *B*. *sinocompressus* and *K*. *tibetica* differed greatly in abundance, height and coverage (S1 Table). Although lower than *K*. *tibetica* in every investigated tussock, *B*. *sinocompressus* appeared to be advantageous at the community scale in abundance and coverage. The gaps between the *K*. *tibetica* tussocks were almost exclusively filled with *B*. *sinocompressus*. Despite the different community views, most of the soil characteristics showed no significant differences between the different sampling locations (S3 Table), indicating the relative homogeneity of the habitat conditions for these two species. Detailed information about the community topology and environmental factors is shown in the supplemental materials. Note that clonality was not taken into consideration in the community census.

### 3.2. Clone assignment and spatial structure

**Fig 3:**
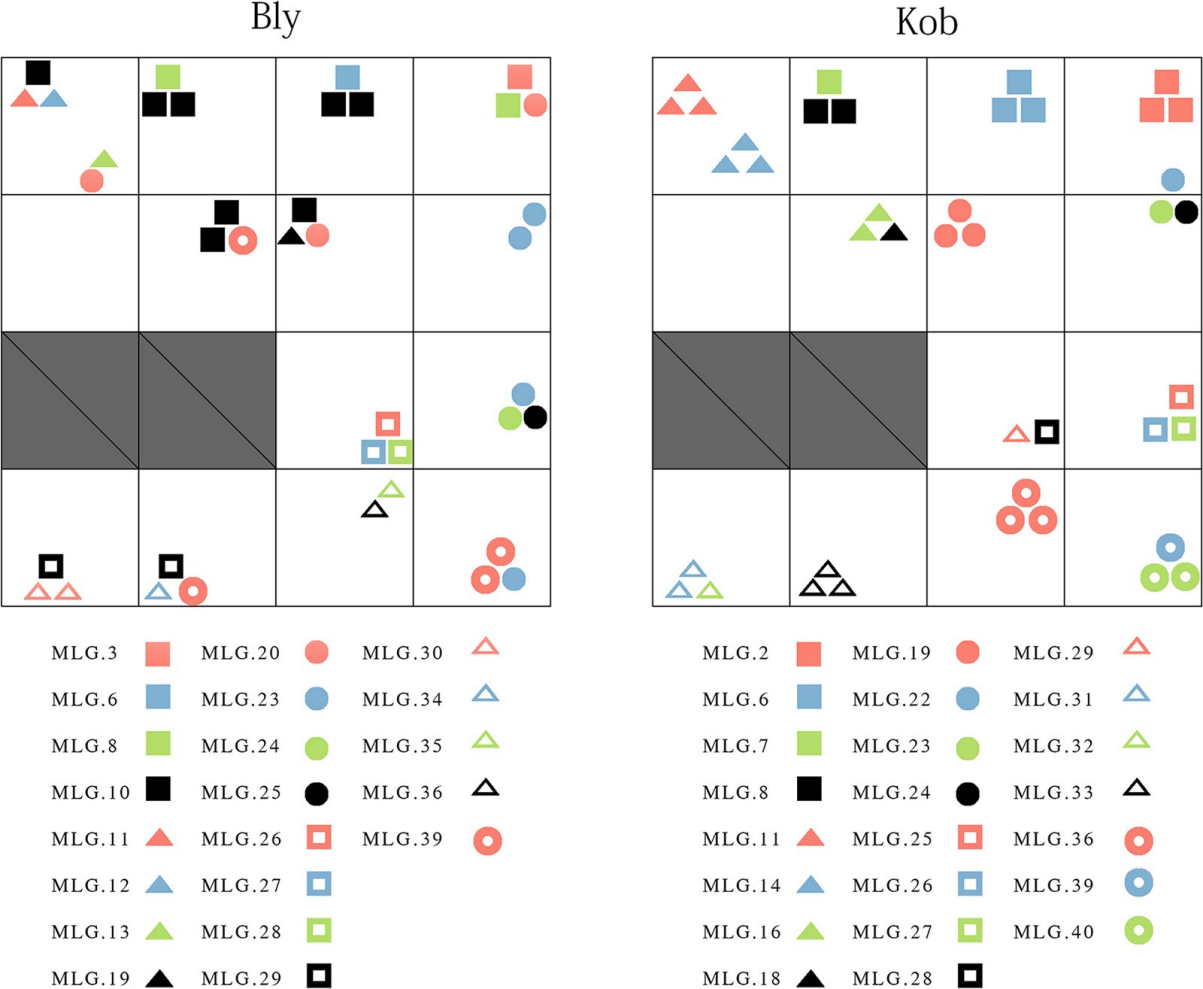
The spatial arrangement of the detected clonal lineages. Each sampled individual was plotted according to clonal assignment and spatial position. The same symbol indicates the same clonal membership. The symbols between species are not relevant. *Blysmus sinocompressus* is abbreviated Bly. *Kobresia tibetica* is abbreviated Kob. The same applies hereafter.

A total of 7710 and 21868 potential SNPs were identified for *B*. *sinocompressus* and *K*. *tibetica* respectively. The average tag number and mapping rate for *B*. *sinocompressus* were (4.90×10^4^, 65.70%), compared to those of *K*. *tibetica* (5.30×10^4^, 66.06%). Detailed results of the sequencing can be found in the supplemental materials (S4 Table, S1 Fig). The potential SNP loci were filtered for further analysis if (1) more than 80% of the sampled individuals could be distinguished at that locus; and (2) the minor allele frequency (MAF) was > 0.01. Finally, 21373 SNPs were used for the clone assignment of *K*. *tibetica*, while 7227 SNPs were used for *B*. *sinocompressus*. A total of 41 genotyped individuals of *K*. *tibetica* were assigned to 23 distinct clonal lineages, while 39 genotyped individuals of *B*. *sinocompressus* were assigned to 21 distinct clonal lineages. Fig 3 shows the spatial arrangement of the identified clonal lineages.

The clone size and diversity results were summarized in Table 1. The clone richness was 0.53 for *K*. *tibetica* and 0.55 for *B*. *sinocompressus*. The clone size was evaluated in terms of ramet size (number of ramets per genet) and spatial size (spatial distance between ramets of the same genet). Although the average ramet sizes of the two species were almost equal, the variation was much higher for *B*. *sinocompressus*. Regarding the physical size, the genets of *B*. *sinocompressus* ranged from 3.10 m to 18.32 m, with an average of 9.85 m. A total of 42.86% (6 out of 14) of the *B*. *sinocompressus* genets were found to have spread among the tussocks. Ramets of MLG 10 were found to have spread over five tussocks. The physical size of *K*. *tibetica* was not available in the spatial distance measure. However, it is reasonable to use the tussock size as the upper limit, as our results showed that all the ramets from the same genet of *K*. *tibetica* were restricted within the tussock. Our results also showed that 50% (7 out of 14) of the *K*. *tibetica* were not monoclonal, indicating that it is not proper to treat the whole tussock as one genetic individual. From the perspective of spatial distribution, clonal lineages of *K*.*tibetica* were more evenly distributed than those of *B*. *sinocompressus*, which is consistent with the more variable size and spreading characteristic of *B*. *sinocompressus*.

**Table 1:** The clone size and diversity information of both species.

Species		Bly	Kob
Richness	N	39	41
	G_g_	21	23
	R	0.55	0.53
Ramet size	min	1	1
	max	8	3
	mean(se)	1.86(0.37)	1.78(0.19)
Spatial size(m)	min	3.10	*
	max	18.32	*
	mean(se)	9.854(0.96)	*
Diversity	G_s_	11.61	18.47
	H	2.76	3.02
	E	0.72	0.90
Distribution	N_mon_	1	7
	N_mul_	13	7
	N_sp_	6	0

N, number of samples; G_g_, number of genets; R, genotypic richness; Ramet size, the amount of ramets per genet; Spatial size, the spatial distance between ramets of the same genet; G_s_, Stoddard and Taylor index; H, Shannon-Wiener index; E, evenness index; N_mon_, number of monoclonal tussocks; N_mul_, number of multiple-clonal tussocks; and N_sp_, number of clones spreading over different tussocks.

* indicates that the spatial size of Kob is not available because all the ramets reside within the tussock.

**Fig 4:**
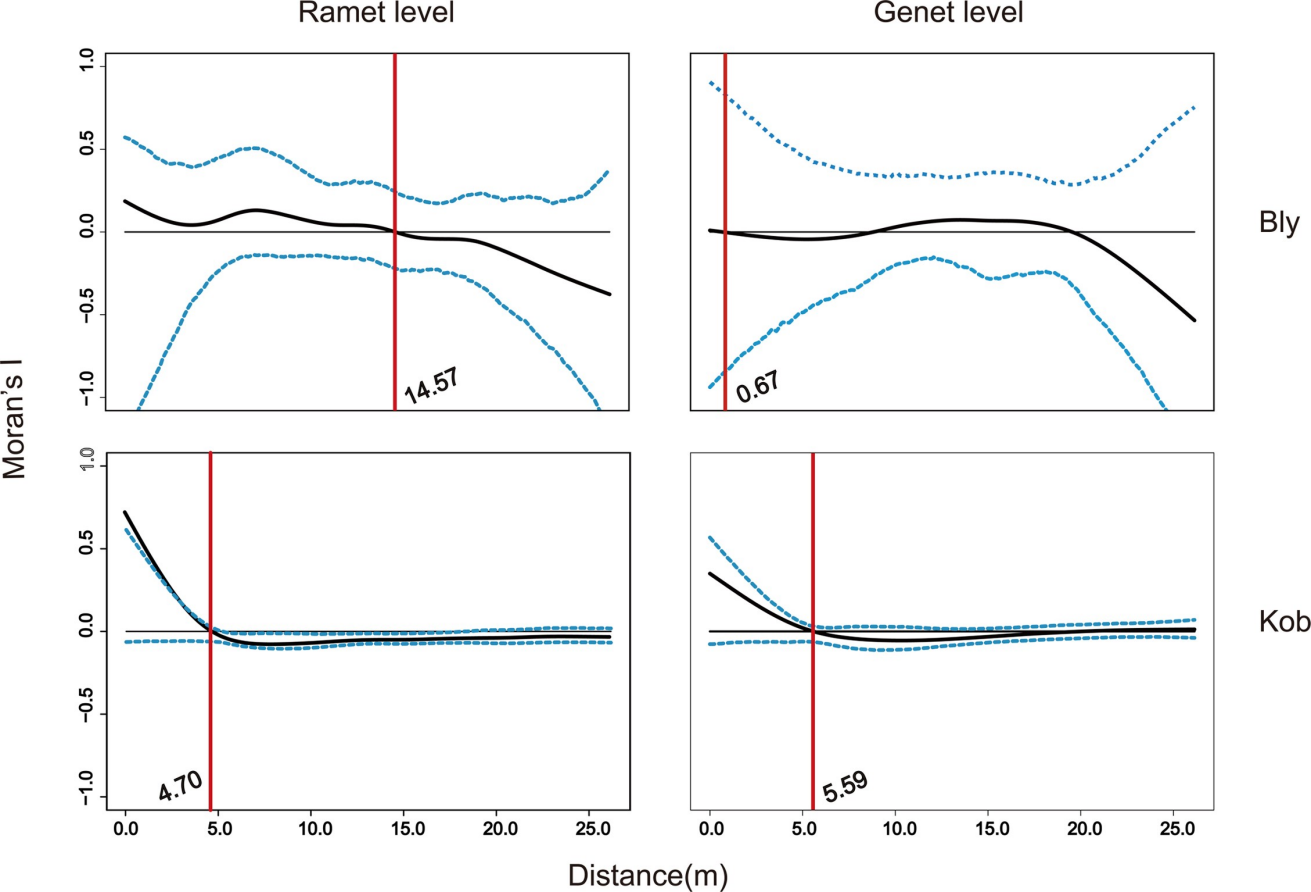
Spatial autocorrelation between kinship and geographic distance for both species at the ramet and genet levels. The dashed blue line envelopes the bootstrap distribution at 1000 resamples. The red line indicates the position of the intercept with a zero-reference line beyond which the genetic relationship is no more similar than that expected by chance alone.

Fig 4 shows the spatial genetic structure of both species. At the ramet level, Moran’s I for *K*. *tibetica* obtained a climax value (Y-intercept = 0.721,) when the distance approached zero. This correlation declined as the distance increased. At a distance of 4.70 m, the correlation intercepted the zero-correlation reference line, indicating that clonality does not affect the genetic structure beyond this spatial distance. The correlation value was reduced (Y-intercept value from 0.721 to 0.350) when no duplication of ramets was included (the genet level), reflecting that the contribution of clonality to fine spatial genetic structure was significant. However, the shape of the simulated curve remained the same, which could be attributed to the clumped distribution of the *K*. *tibetica* ramets. For *B*. *sinocompressus*, the spatial genetic structure was relatively weak, even at the ramet level (Y-intercept = 0.186). Clonality affected the spatial genetic structure within 14.57 m, which is approximately the biggest spatial size of the detected clones.

### 3.3. Genetic diversity and evolutionary relationship

A total of 7256 SNP loci were ascertained for *B*. *sinocompressus*, while 19501 SNP loci were ascertained for *K*.*tibetica*, indicating a higher level of genetic variability in *K*.*tibetica*. (Table 2). However, the average allele number at each locus was almost the same for both species, which could be attributed to the prevalence of biallelic loci in the SNP markers. The polymorphism information content (PIC) value showed that *B*. *sinocompressus* had a moderate polymorphism (0.264), while *K*. *tibetica* had a low polymorphism (0.132). Considering that the PIC value takes allele frequency into account, this result reflected that many rare alleles were preserved in *K*.*tibetica*. The expected heterozygosity (He) for *B*. *sinocompressus* was higher than that of *K*.*tibetica*, but the observed heterozygosity (Ho) showed the opposite trend. The inbreeding coefficient (F_IS_) for *B*. *sinocompressus* was 0.559, which was five times greater than that of *K*.*tibetica*(0.093). These results showed that the detected genetic diversity of *K*. *tibetica* was higher than that of *B*. *sinocompressus*, and nonrandom mating was much more common for *B*. *sinocompressus*, which could be attributed to selfing within flower or geitonogamous pollination between ramets.

**Table 2:** Summary of the genetic diversity information and inbreeding levels for both species.

Species	Loci information			Heterozygosity		Inbreeding
	n	N_A_	PIC	H_e_	H_o_	F_IS_
Bly	7256	2.063(0.242)	0.264(0.145)	0.338	0.081	0.559
Kob	19501	2.023(0.207)	0.132(0.108)	0.153	0.143	0.093

n, number of loci; N_A_, average allele number per loci with SD in parenthesis; PIC, average polymorphism information content value; H_e_, expected heterozygosity; H_o_, observed heterozygosity; and F_IS_, inbreeding coefficients.

**Fig 5:**
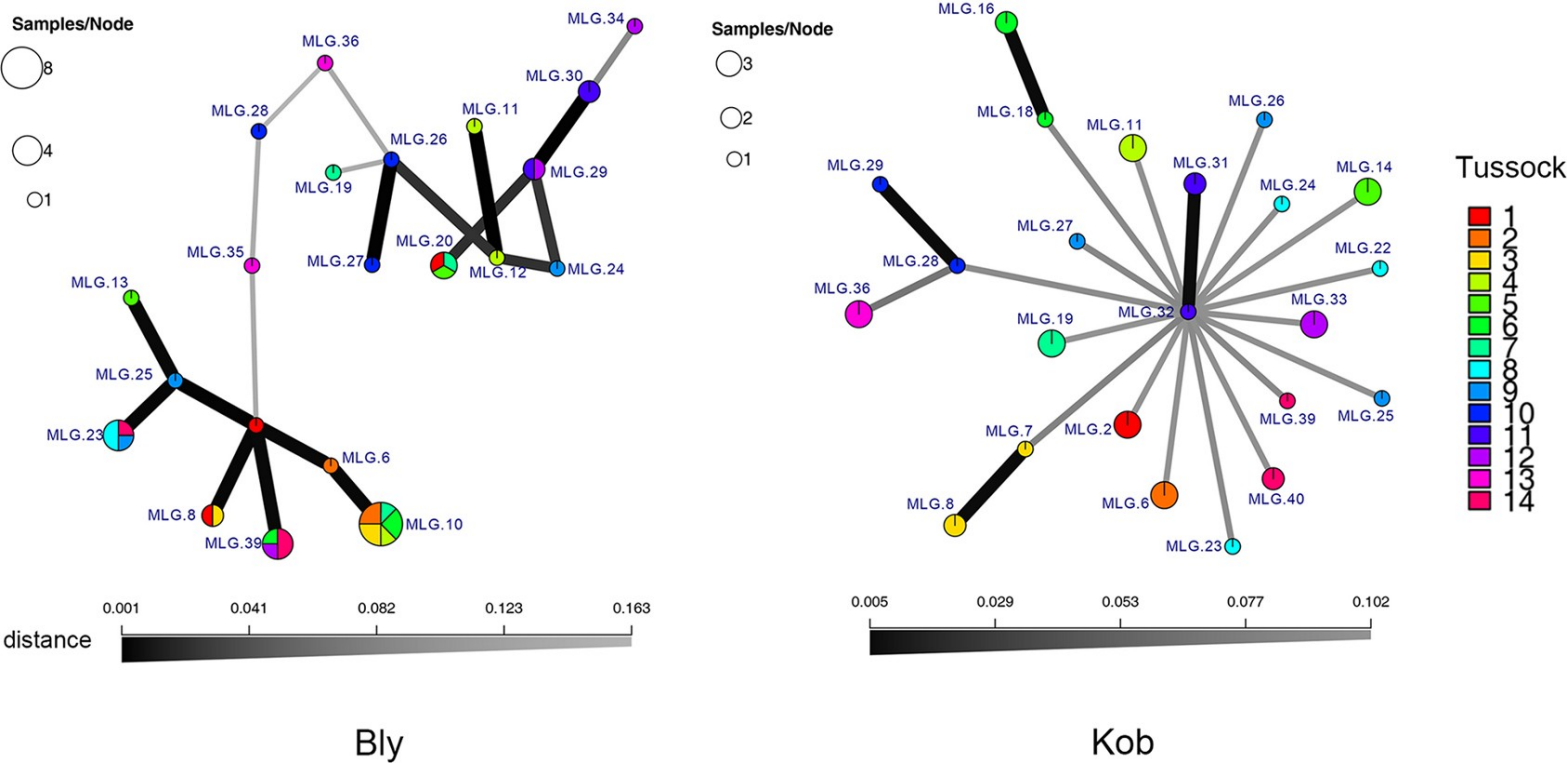
Minimum spanning tree (MSN) showing the evolutionary relationship of the clonal lineages. The size of a node is proportional to the number of assigned ramets. Color represents the tussock where the samples were taken. The wider and darker lines indicate a relatively higher relatedness. The position of the nodes is arbitrary.

The results of the MSN showed contrasting patterns for the MLGs of these two species (Fig 5). The MLGs of *B*. *sinocompressus* were mainly clustered into two groups, indicating that most of the MLGs were genetically closely related. The ramets assigned to a specific MLG of *B*. *sinocompressus* could come from different tussocks. The number of ramets per MLG was variable. Few large MLGs are composed of more than four ramets (MLG 10). In contrast, the MLGs of *K*.*tibetica* were genetically dispersed with no detected structure. The number of ramets per MLG was relatively stable, mainly 2–3 ramets. All the ramets assigned to a specific MLG came from the same sampled tussock. The AMOVA results (Table 3) showed the origin of the variance for both species. For *K*. *tibetica*, variance among tussocks contributed 78.51% to the total amount of variance, while variance within tussock explained the remaining 21.49%. For *B*. *sinocompressus*, most of the variance was within a tussock (71.37%). Both species had a positive *p* value at a significance level of 0.05, but the effect for *K*. *tibetica* tended to be more significant.

**Table 3:** Summary of the analysis of molecular variance (AMOVA) of both species showing the origin of the variance.

Species	Source	df	SSD	MSD	Variance	% total	*p*
Kob	Among tussocks	13	80904.69	6223.44	1944.52	78.51	<0.001
	Within a tussock	27	14368.17	532.15	532.15	21.49	
	Total	40	95272.85	2381.82			
Bly	Among tussocks	13	40434.1	3110.32	589.75	28.63	0.048
	Within a tussock	25	36754.33	1470.17	1470.17	71.37	
	Total	38	77188.44	2031.28			

## 4. Discussion

The alpine wetland vegetation of the Qinghai-Tibet Plateau is often characterized by the prevalence of two hygrophytes: *Blysmus sinocompressus* and *Kobresia tibetica*. The latter species is a tussock-building perennial, while the former species acts similar to a contemporary successional species during wetland deterioration. Based on the SNPs identified by 2b-RAD sequencing, we contribute the first available clonal structure and the fine-scale genetic structure of these two species, which could aid in understanding the process of degrading succession in alpine tussock swamps.

### 4.1 Clonal structure and spatial patterns

The size and spatial arrangement of genets are of fundamental importance in a clonal population because they affect the mating opportunities of individuals and provide the basis for long-term preservation and expansion [10, 11, 13]. Our results show that *K*. *tibetica* has a phalanx growth form. The clonal diversity of *K*. *tibetica* (R = 0.53, H = 3.02) is similar to *K*. *pygmea* (R = 0.41, H = 3.02) [23]. All the ramets of a specific clone are restricted within a tussock, which reflects the production of short rhizomes described in previous studies [26]. This phalanx growth form is also supported by the steep autocorrelation curve (Fig 4), which shows that the spatial range limit of clonality on genetic structure is 4.70 m. However, approximately 50% (7 out of 14) of the tussocks host more than one MLG, indicating that multiple clones may coexist in one tussock. This may be attributed to seedling recruitment from the seed bank, as tussocks tend to promote the sexual reproduction of inhabitant species [15, 46, 47]. Our results also imply that it may be incorrect to treat all the individuals from a clumped cluster as one clone, which is consistent with the findings for *Carex sempervirens*[18].

As for *B*. *sinocompressus*, the population tends to be composed of intermingled genets with a guerilla growth form. The largest detected clone size was 18.32 m (Table 1), which complies with the record of Hu *et al*. [25] that *B*. *sinocompressus* is a far creeping species. The AMOVA results also show that the major portion of the variation is distributed within a tussock for *B*. *sinocompressus* (Table 3), which is in accordance with the expansion of genets among different tussocks (Table 1). However, the clone size distribution is uneven. The fluctuation in clone size was greater for *B*. *sinocompressus* than that of *K*. *tibetica* (Table 1, Fig 3). There are two causes for a large variability in clone size: (1) clones of different sizes may reflect successive events of seedling recruitment, ranging from old and large genets to recently established, small genets; and (2) small clones could also represent the remains of formerly larger clones that partially died [23]. Considering the recent emergence of *B*. *sinocompressus* in the succession, the first explanation is plausible. Additionally, *B*. *sinocompressus* had similar clone richness but lower diversity (R = 0.55) compared with *K*. *tibetica*, indicating that sexual reproduction may be nearly equal in both species. These results imply that clonal growth in the guerilla form may enhance clone expansion and consolidate the advantage of *B*. *sinocompressus* in the community, which is a strategy favored by clonal plants in optimal environments [48].

### 4.2 Mating patterns and the succession process

Although clonal growth could increase the probability of a within-clone movement of gametes that may lead to a fitness cost (e.g., self-fertilized offspring), some studies have shown that this effect is contextual on the interaction between the spatial arrangement of clones and their biological traits [12, 13]. Both species are wind pollinated and have mixed reproductive modes, but they have contrasting inbreeding levels (Table 2). The low inbreeding level in *K*. *tibetica* implies the following: (1) pollen flow among the tussocks of *K*. *tibetica* is not hindered by spatial separation; and (2) geitonogamous selfing within tussock is effectively avoided. The mechanism for inbreeding avoidance seems to be complex. One possible reason is dichogamous flower development (dichogamy). It has been reported that the synchronization of sexual function among ramets of a clone (i.e., ramets of the same clone present the same sexual phase at a given time) could limit inter-ramet geitonogamy[49]. Cruden reported the prevalence of this phenomenon in 37 diverse angiosperm families, including many rhizomatous clonal perennials (e.g., *Typha*, *Sparganium*, *Scirpus*) [50]. Alternatively, self-compatibility or postzygotic barriers may also contribute to the inhibition of inbreeding [9]. Further research effort is needed, as the information about the breeding system of *Kobresia* is very limited. The higher inbreeding level of *B*. *sinocompressus* could be explained by the effect of clone expansion. As clone size increases, outcrossing pollen to disperse across different clones becomes more difficult. The low height of *B*. *sinocompressus* may also contribute to the difficulty because winds tend to be weakened on the lower surface of the microtopography [51]. Our results show that the phalanx growth form is not necessarily prone to inbreeding. The effect of clonal structure on mating pattern tends to be contextual on both biotic and abiotic factors.

Many plants utilize a combination of sexual and asexual reproduction, and the balance between these strategies varies widely within and among taxa [52]. Facultative sexual reproduction in clonal plants plays an important role in maintaining genetic diversity and evolutionary potential. Thus, the genetic relatedness of original genets could influence their population viability in the long term. In wetlands, the “opportunity window” for succession often presents itself when flood retreats and seedlings emerge rapidly due to dormancy relief [53, 54]. This is also the case for *B*. *sinocompressus*, which colonizes the gaps between *K*. *tibetica* tussocks as the retained water disappears. However, most of the *B*. *sinocompressus* genets were more closely related and assigned to two clusters (Fig 5), indicating that they are probable inbred offspring of a few old genets. Consequently, the evolutionary potential is constrained by the ancestral genets, resulting in a deficiency of genetic diversity. Zhao *et al*. have found that the input of genets from seedlings matters in determining the genetic diversity of clonal plants [26], which is consistent with our results. Although the combined effect of clonal growth and seedlings enables temporary advantages, *B*. *sinocompressus* may be vulnerable to future disturbances, such as grazing and degradation [25, 55]. In contrast, genets of *K*. *tibetica* are more evolutionarily separated and present a high level of variability. Previous studies have indicated that even low rates of seedling recruitment are sufficient for maintaining high levels of genetic diversity [9, 10]. As isolation among tussocks tends to be enhanced during degradation, the coexistence of genetically distant genets within tussocks is of vital importance in providing the necessary levels of gene flow. Generally, our results support the view that the genetic load of the original genets explains the high genetic diversity of *Kobresia*. The remaining tussocks in degrading wetlands stand as valuable genetic relics of a former well-developed *K*. *tibetica* meadow, which is worthy of more conservation or restoration attention.

## 5. Conclusions

In summary, we reveal the clonal structure and fine-scale genetic structure of two alpine plants (*Kobresia tibetica* and *Blysmus sinocompressus*) in the context of wetland succession. The tussock builder *K*. *tibetica* has a phalanx growth form, but different genets can coexist within the same tussock. It is not proper to treat a tussock as one genetic individual. *B*. *sinocompressus* has a guerilla growth form and considerable variability in clone size, indicating a successive recruitment from seedlings. Our results demonstrate that the combination of clonal growth and seedlings contributes to the advantage of *B*. *sinocompressus* at the early stage of degradation. Nevertheless, most genets of *B*. *sinocompressus* tend to be inbred offspring of a few old genets, resulting in their deficient evolutionary potential. In contrast, genets of *K*. *tibetica* present inbreeding avoidance despite the close placement of their ramets, indicating that tussocks are valuable genetic relics worthy of conservation attention. It is important to recognize that this study only assessed one community on a fine scale, and the underlying mechanism is not clear due to the lack of information on the inbreeding systems of both species. Further research efforts are needed to elucidate the gene flow processes of both species in various habitat conditions, especially with the knowledge of pollination biology and the degrees of self-compatibility.

## Supporting information

S1 Table Community demography showing the abundance, coverage and height of both species. (XLS)

S2 Table Information regarding the adaptors and primers used in library construction. (XLS)

S3 Table *t*-test results of eight types of soil properties demonstrating the differences in the microhabitats. (XLS)

S4 Table Summary of the results of the enzyme digestion and mapping for each sampled individual. (XLS)

S1 Fig The overall sequencing quality showing the distribution and quality of the acquired bases. (PDF)
