# Supplementary figures and images for: Contrasting fine-scale genetic structure of two sympatric clonal plants in an alpine swampy meadow featured by tussocks
Source: PLoS One. 2018 Dec 21;13(12):e0209572. doi: 10.1371/journal.pone.0209572 (PMC6303067; doi:10.1371/journal.pone.0209572)

**A****Base percentage composition**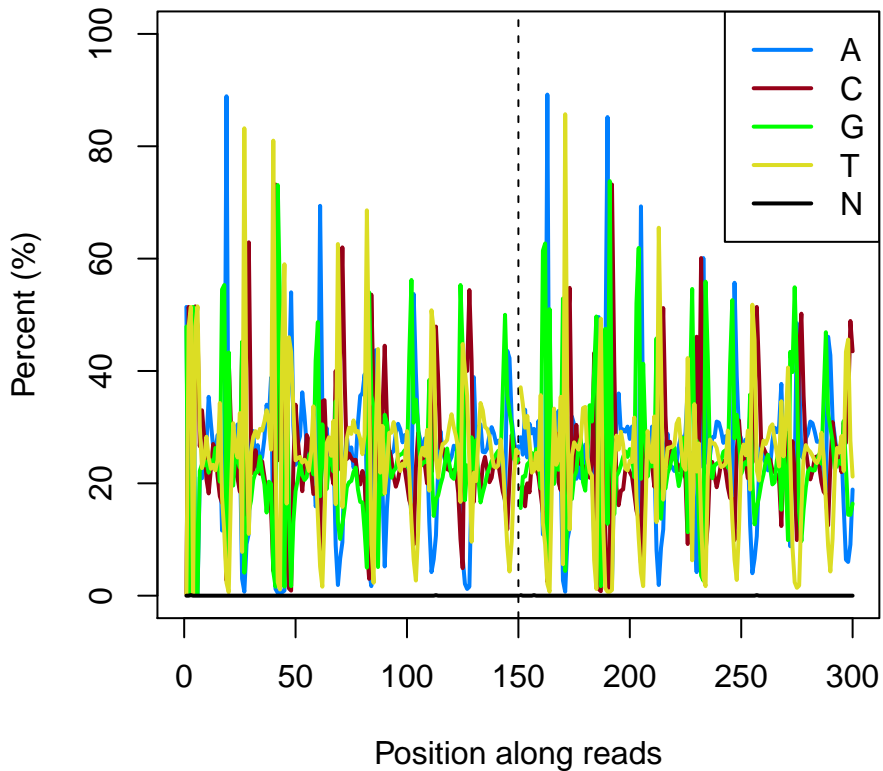**B****Distribution of qualities**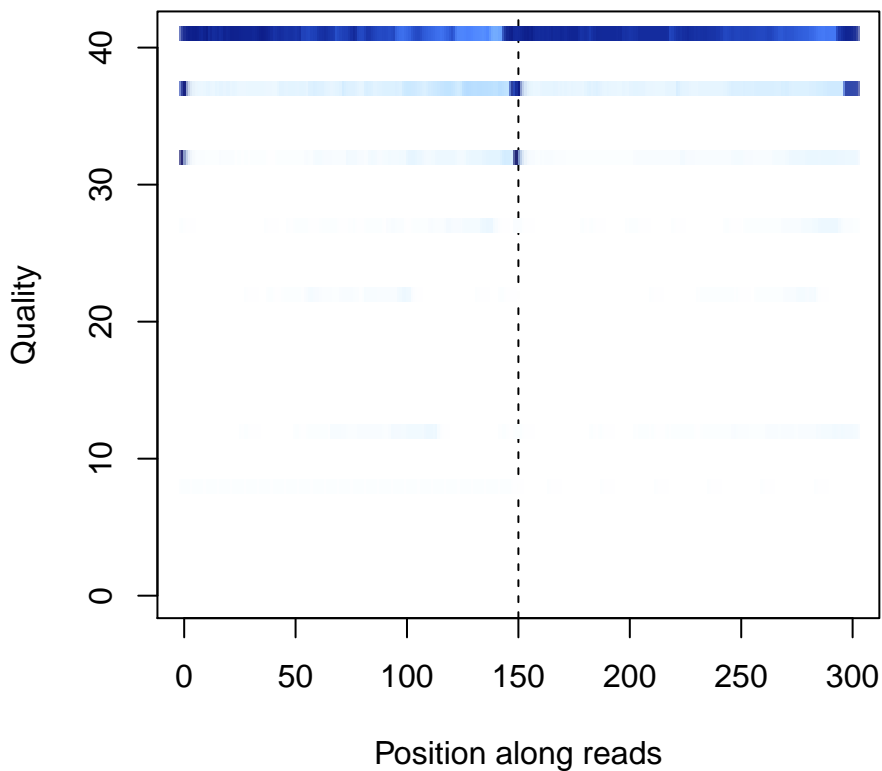

Supplement: S1 Fig — (PDF) [file pone.0209572.s005.pdf]
